# The Efficacy of High-Flow Nasal Cannula (HFNC) Treatment in Patients with Chronic Type II Respiratory Failure Secondary to COPD

**DOI:** 10.3390/jcm15051924

**Published:** 2026-03-03

**Authors:** Raffaella Pagliaro, Vittorio Simeon, Luca Notizia, Stefania Arena, Domenica Francesca Mariniello, Giulia Maria Stella, Andrea Bianco, Fabio Perrotta, Luigi Aronne

**Affiliations:** 1 Department of Translational Medical Sciences, University of Campania “Luigi Vanvitelli”, 81100 Caserta, Italy; raffaella.pagliaro@studenti.unicampania.it (R.P.); luca.notizia@gmail.com (L.N.); nikamariniello93@gmail.com (D.F.M.); andrea.bianco@unicampania.it (A.B.); luigi.aronne@gmail.com (L.A.); 2 Clinic of Respiratory Diseases “Vanvitelli”, A.O. dei Colli, Monaldi Hospital, 80131 Naples, Italy; 3 Department of Mental and Physical Health and Preventive Medicine, University of Campania “Luigi Vanvitelli”, 80138 Naples, Italy; vittorio.simeon@unicampania.it (V.S.); stefania.arena@unicampania.it (S.A.); 4 Department of Internal Medicine and Medical Therapeutics, Medical School, University of Pavia, 27100 Pavia, Italy; g.stella@smatteo.pv.it; 5 Unit of Respiratory Diseases, Cardiothoracic and Vascular Department, IRCCS Policlinico San Matteo, 27100 Pavia, Italy

**Keywords:** COPD, HFNC, chronic hypoxemic–hypercapnic respiratory failure, AECOPD, reduction in PaCO_2_

## Abstract

**Background:** The use of HFNC (High Flow Nasal Cannula) in the management of acute respiratory failure has been fully established in clinical practice. Conversely, less data is available supporting its use in chronic hypoxemic–hypercapnic respiratory failure. The aim of the present study is to evaluate the efficacy of HFNC in chronic hypercapnic respiratory failure associated with stable COPD. **Methods:** In this retrospective single-center longitudinal observational study, 40 patients treated with HFNC at home followed at the COPD Clinic of Respiratory Diseases (University of Campania L. Vanvitelli Monaldi Hospital, Naples) were included. All patients are re-assessed at our clinic at T0, T3, T6 and T12 months through functional respiratory tests and blood gas analysis. **Results:** After 12 months, significant reductions in pCO_2_ (arterial partial pressure of carbon dioxide) (from 58.5 to 48.0 mmHg) and lactates (from 1.60 to 0.90 mmol/L) were observed, and MIP and MEP improved significantly. Patients receiving HFNC flows ≥50 L/min experienced greater reductions in pCO_2_ and fewer exacerbations. Multivariate analysis identified HFNC flow rate (*p* = 0.0046), hours of use/day (*p* = 0.0157), lactate levels (*p* = 0.0301), and FEV_1_ (forced expiratory volume in 1 s) (*p* = 0.0491) as independent predictors of reduction in PaCO_2_. Higher BMI and greater airway obstruction were associated with a reduced response. **Conclusions:** Treatment with HFNC represents a reasonable therapeutic choice to reduce AEs-COPD and reduce PaCO_2_ and lactates in stable COPD patients.

## 1. Introduction

Chronic Obstructive Pulmonary Disease (COPD) represents a progressive respiratory condition characterized by chronic airflow limitation, increased inflammation in the airways [1,2] and chronic respiratory symptoms such as dyspnea, cough, and sputum production [3,4]. In general, COPD is considered one of the major causes of morbidity and mortality worldwide [5,6]. This condition gradually results in chronic respiratory failure, which can cause hypoxia and hypercapnia; both complications are linked to poor outcomes of these patients [7]. In addition, common risk factors in COPD may lead to comorbidities, which include lung cancer, cardiovascular and metabolic diseases, and muscle wasting [8,9,10,11,12]. However, in COPD patients, respiratory failure can occur as acute, chronic or acute-on-chronic failure. Acute exacerbation of COPD (AECOPD) is due to a worsening of the disease with an increase in symptoms such as cough, sputum production and dyspnea that worsen in <14 days [13]. The impact of AECOPD can vary widely, ranging from a temporary condition to a serious progression of respiratory failure [13,14,15]. The treatment for exacerbations primarily included administration of bronchodilators, corticosteroids and antibiotics [16]. In the most severe cases, respiratory support ranging from supplemental oxygen to invasive and non-invasive ventilation may be required [17]. In the chronic setting, patients often showed rapid shallow breathing [18]. Long-term oxygen therapy (LTOT) has been shown to significantly reduce mortality [19]. In this context, non-invasive ventilation (NIV) is considered the standard treatment modality for hypercapnic respiratory failure, particularly when complicated by respiratory acidosis [18]. Unfortunately, there are major drawbacks associated with the use of NIV, including facial skin breakdown, claustrophobia, gastric inflation, air leaks and patient–ventilator asynchronies, being some of the most frequent causes of poor compliance and treatment failure [20].

The high-flow nasal cannula (HFNC) is a non-invasive respiratory support that delivers heated, humidified gas with adjustable oxygen fraction via nasal cannula at high flow rates (up to 60 L per minute) [21]. The treatment with HFNC involves the supply of gas mixtures containing varying concentrations of oxygen (from 21 to 100%), moistened, heated and administered at a flow that counterbalances or overcomes the patient’s peak inspiratory flow (PIF) [22]. HFNC has pleiotropic effects, creating a low level of positive end-expiratory pressure (PEEP), washing out of anatomical dead space in the upper airways, decreasing inspiratory effort, actual delivery of the chosen FiO_2_, fluidization of secretions and improving airway clearance. The improvement in carbon dioxide retention is linked to an increase in tidal volume and a decrease in respiratory rate that contributes to improved alveolar ventilation [23]. In a recent meta-analysis, Lu et al. detected that HFNC is not inferior to NIV in decreasing PaCO_2_, improving PaO_2_ and SpO_2_ and reducing inspiratory muscle effort [24]. However, in patients with COPD and chronic hypercapnia, HFNC appears to reduce acute exacerbations and may decrease hospitalizations compared with standard care [25]. Therefore, HFNC represents a viable therapeutic option for patients with stable hypercapnic COPD who have a history of exacerbations [26,27]. HFNC significantly improves quality of life. These devices are used in the intensive and sub-intensive care of adults for the treatment of hypoxemic respiratory failure and for weaning them off the ventilator [14,28]. However, in patients with COPD and stable hypercapnia, home HFNC therapy has been shown to lower PaCO_2_ levels, improve the quality of life, reduce the frequency of exacerbations and hospital admissions and enhance airway washout, leading to a reduction in functional dead space [29]. The long-term benefit of domiciliary HFNC on patients with stable COPD has been explored, but there are still few literature data evaluating its effects in long-term home treatment in COPD patients [30,31].

The aim of this study was to demonstrate that the use of HFNC at home in patients with chronic hypercapnic respiratory failure secondary to COPD allowed for a reduction in the values of PaCO_2_ in the arterial blood and consequently a reduction in the annual exacerbation rate by evaluating the number of hours of use per day.

## 2. Materials and Methods

We performed a retrospective single-center longitudinal observational study conducted at the COPD Clinic of Respiratory Diseases, University of Campania Luigi Vanvitelli Monaldi Hospital, Naples. We enrolled COPD patients with chronic respiratory failure who were treated with HFNC and did not require NIV treatment. These patients received HNFC in addition to standard medical therapy and oxygen supplementation and were followed up as a part of their routine clinical care.

### 2.1. Inclusion Criteria

The inclusion criteria were: patients with diagnosis of COPD based on spirometry according to GOLD recommendation [1]; age ≥ 40 years; significant history of smoking (current or former smokers with a pack years greater/equal to 20); type II respiratory failure requiring long-term oxygen supplementation (PO_2_ < 60 mmHg and pCO_2_ greater/equal to 45 mmHg in at least 2 measurements prior to T0); bronchodilator therapy with ICS + LABA + LAMA with single or multiple inhalers; patients with COPD stability in triple bronchodilators for a minimum of 8 weeks; prescription for home treatment with HFNC using the MyAirvo2 device from Fisher & Paykel for at least 12 h per day with varying oxygen enrichment; presence of a stable clinical condition with no exacerbations for at least 12 weeks prior to study enrollment.

### 2.2. Exclusion Criteria

The exclusion criteria were: history of respiratory sleep disorders requiring night ventilation with CPAP/NIV; concomitant other lung disease such as Interstitial Pulmonary Diseases or Bronchiectasis; diagnosis of cancer in the previous 5 years (except non-melanomatous skin cancer); use of systemic glucocorticosteroids and/or antibiotics within the previous 12 weeks preceding the time of enrollment (T0); documented worsening of heart failure requiring changes in routine therapy in the 12 weeks preceding T0; therapy changes for COPD in the 12 weeks preceding T0 (including changes in bronchodilator therapy, addition or discontinuation of PDE-i, theophylline, macrolides); other conditions responsible for global respiratory failure (Type II) such as neuromuscular diseases or chest wall disorders; inability to sign informed consent.

### 2.3. HFNC Intervention and Follow-Up

The entire study population was followed up in the outpatient clinic with a prescription for optimal inhalation therapy according to GOLD recommendations and long-term oxygen therapy (24 h/day), using HFNC through the MyAirvo2^®^ device (Fisher & Paykel Healthcare, Auckland, New Zealand), for at least 12 h/day, including non-continuous use, but preferably at night. Although patients had chronic compensated hypercapnia, none had a clinical indication for long-term NIV at the time of HFNC prescription. The device was set with parameters aimed at maintaining an SpO_2_ target between 90% and 93%, adjusting the FiO_2_ to the lowest possible level, using the maximum flow tolerated by the patient (with the goal of ensuring a minimum flow of over 35 L/min), and the maximum temperature tolerated by the patient. COPD patients were recruited on an outpatient basis at time 0 (recruitment period between October 2022 and October 2023). In the screening phase of our study, patients underwent preliminary tests: blood gas analysis in ambient air (pH, pCO_2_, PO_2_, HCO_3_^−^, lactates, P/F), global spirometry (FEV_1_, FEV_1_%, FVC, FVC%, RV, RV%), alveolar-capillary CO diffusion (DLCO and DLCO%), maximum inspiratory pressure (MIP and MIP%) and maximum expiratory pressure (MEP and MEP%), evaluation of the number and quality of exacerbations in the previous year. Patients were also given specific questionnaires: Modified British Medical Research Council Questionnaire (mMRC) and COPD Assessment Test (CAT). The follow-up visits were scheduled at 3 months (T3), 6 months (T6) and 12 months (T12). At each visit, we assessed the COPD treatment regimen, hospital admissions, any moderate exacerbations of the disease, and adherence to the use of HFNC, evaluating the total usage time (in hours/day) and the settings on the device (temperature, flow, and FiO_2_). We also classified the exacerbations into mild, moderate and severe according to current recommendations [32,33]. The total number of exacerbations during the observation period and their severity were also evaluated during the final visit. The total observation and follow-up period lasted 12 months (ending in October 2024).

### 2.4. Ethics

Patients were treated according to good clinical practice and the collection of data in no way affects the patient’s clinical care. Patients were enrolled after signing informed consent and reading the explanatory information on the study. The Observational Clinical Study was conducted in accordance with the ethical principles of the Helsinki Declaration. The local ethical committee approved the present study with the number 10486/2024.

### 2.5. Endpoint

The primary endpoint of the study was to observe and demonstrate whether the use of HFNCs at home in patients with chronic hypercapnic respiratory failure secondary to COPD allowed them to obtain a stable reduction in PaCO_2_ values in the arterial blood over a 12-month follow-up period. Secondary endpoints included the evaluation of changes in blood lactate levels as an indirect marker of reduced work of breathing, improvements in MIP and MEP, and the reduction in the annual rate of COPD exacerbations compared with the year preceding the initiation of HFNC therapy as an indicator of clinical disease stability. In addition, secondary analyses aimed to identify clinical and treatment-related factors influencing the response to HFNC, including body mass index, delivered flow rate, and daily duration of HFNC use.

### 2.6. Statistical Analysis

Continuous variables are reported as median and interquartile range (IQR), while categorical variables are presented as absolute numbers and percentages. Comparisons between baseline and follow-up time points (T3, T6, and T12) were conducted using the Wilcoxon signed-rank test for within-subject repeated measures to evaluate changes in arterial blood gas values (PaCO_2_, lactates), pulmonary function parameters, and clinical outcomes, with *p*-values adjusted for multiple comparisons using the Benjamini–Hochberg procedure. Multivariate linear regression models were developed to assess the independent association of HFNC-related variables (flow rate, daily usage hours) and baseline patient characteristics, including reduction in PaCO_2_ and lactate levels, changes in MIP and MEP, FEV_1_ variation, and number of exacerbations at 12 months. A *p*-value of <0.05 was considered statistically significant. All analyses were performed using statistical software STATA v16 (StataCorp. 2019. College Station, TX, USA: StataCorp LLC).

## 3. Results

The baseline characteristics of the study population are reported in Table 1. 40 patients (65% males) with chronic respiratory failure secondary to COPD were included in our study. The study population had a median age of 68 years (IQR: 62–76), and the median BMI was 28.0 kg/m^2^ (IQR: 26.0–29.0). Occupational exposure was reported in 15 patients, while allergies were reported in 15 patients. Regarding comorbidities, systemic arterial hypertension was the most prevalent condition (83%), followed by chronic renal failure (63%), dyslipidemia (50%), chronic heart failure (48%) and diabetes mellitus (33%). Most patients were receiving cardiovascular and metabolic therapies, including ACE inhibitors and PPIs (75% each), beta blockers and statins (58% each), and antiplatelet agents (40%). All patients were former smokers with a median of pack years of approximately 34.63 (range 20–50). They were treated for COPD with triple inhaler therapy, including LABA-LAMA-ICS with a single inhaler in 88% of cases, while 12% with multiple inhalers. Pulmonary function tests (forced expiratory volume in 1 s (FEV_1_), forced vital capacity (FVC), total lung capacity (TLC), residual volume (RV), diffusing capacity of the lungs for carbon monoxide (DLCO), maximal inspiratory pressure (MIP), maximal expiratory pressure (MEP)) were evaluated and are shown in Table 1.

**Figure 1:**
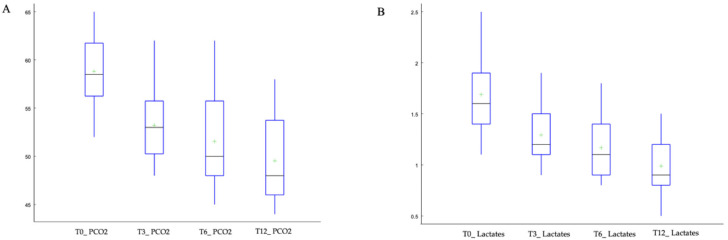
Trends in pCO_2_ levels (**A**) and lactate levels (**B**) over 12-month follow-up period. Values are shown at baseline (T0), after 3 months (T3), 6 months (T6) and 12 months (T12). Both parameters demonstrated improvements by 3 months, which were maintained throughout the follow-up period. Green plus signs indicate outliers.

Moreover, we divided the study population into two groups based on the HFNC flow setting: low-flow (35–45 L/min) and high-flow (50–60 L/min). Specifically, 13 patients were managed with a flow rate < 50 L/min, while 27 patients tolerated a flow rate ≥ 50 L/min. Over the 12-month follow-up period, we observed a statistically significant reduction in pCO_2_ and lactate levels across the study population. Both parameters showed significant improvements after 3 months, which were maintained at 6 and 12 months, with an overall median reduction of approximately 10 mmHg by T12 (Figure 1A). In particular, pCO_2_ values showed a significant reduction, with a median value decreasing from 58.5 (56.5, 61.5) mmHg to 53.0 (50.5, 55.5) mmHg. This downward trend continued over time, reaching a median of 48.0 (45.5, 51.5) mmHg, with the lowest observed value being 44 mmHg. A similar trend was observed for lactate levels (Figure 1B), which showed a statistically significant reduction at T3. The median value decreased from 1.60 (1.40, 1.90) mmol/L at baseline to 1.20 (1.10, 1.50) mmol/L at T3, and this reduction remained stable through T12 at 0.90 (0.80, 1.20).

**Figure 2:**
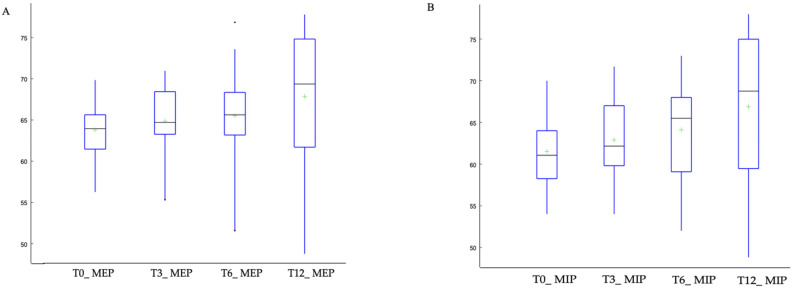
Trends in maximum expiratory pressure (MEP) (**A**) and maximum inspiratory pressure (MIP) (**B**) over the 12-month follow-up period. Values are shown at baseline (T0) and after 3 (T3), 6 (T6), and 12 months (T12). Green plus signs indicate outliers.

At the same time, we observed a significant increase in MEP as early as 3 months (T3) (Figure 2A) and in MIP at 12 months (T12) (Figure 2B). Specifically, the median MIP increased from 61 (59, 64) at baseline to 69 (60, 75) at T12, while the median MEP value increased from 74 (72, 76) to 80 (73, 86) for MEP over the same period.

Pairwise comparisons confirmed that reductions in PaCO_2_ and lactate levels were significant from 3 months and remained stable through 12 months, while respiratory muscle strength improved earlier for MEP and later for MIP (Table 2).

After 12 months of home-based HFNC treatment, multivariate analysis (Table 3) revealed a statistically significant correlation between treatment response—measured by changes in FEV_1_, pCO_2_ and lactate levels—and the patient’s body weight. These findings suggest that HFNC therapy is more effective in achieving a reduction in PaCO_2_ in patients with lower BMI, while those with higher BMI tend to respond less favorably.

Moreover, multivariate analysis included the evaluation of HFNC daily use as the dependent variable (Table 3). At 12 months (T12), a statistically significant correlation was found between the number of hours of HFNC use and both the reduction in pCO_2_ and the increase in MIP values. MIP values remained stable at 3 months (T3), improving at 6 months (T6), and became statistically significant by T12, while pCO_2_ levels showed a significant reduction already at T3. Increased HFNC usage time was strongly associated with a lower number of exacerbations. Descriptive analysis of pCO_2_ levels showed a median decrease from 58.5 (56.5, 61.5) mmHg at baseline to 48.0 (45.5, 51.5) mmHg at T12. According to the regression model, only FEV_1_% and HFNC flow rate were significant predictors of this change (*p* < 0.05). In particular, higher HFNC flow rates were associated with greater reductions in pCO_2_, while higher baseline FEV_1_% was associated with smaller reductions (β = −0.10, *p* = 0.014). These findings suggest that HFNC flow plays a critical role in reducing hypercapnia over time, while baseline lung function modulates the extent of this effect.

Furthermore, patients who used HFNC for more hours per day experienced fewer exacerbations (Table 4), whereas those with lower daily usage and lower tolerated flow rates had more frequent exacerbations, particularly those unable to tolerate a flow ≥ 50 L/min. It is important to note that the adherence to HFNC therapy improved progressively throughout the study observational period. Even patients who initially used HFNC for fewer hours per day showed increased compliance over time.

However, a regression analysis with stepwise selection identified HFNC flow rate as the significant predictor of the total number of exacerbations over 12 months (coefficient = −0.128; *p* < 0.001). Specifically, for every 1 L/min increase in flow rate, the expected number of exacerbations decreased by approximately 12%. None of the other clinical variables initially included in the model (FEV_1_%, BMI, pCO_2_, RV%) reached statistical significance.

Finally, a multivariate analysis was performed to identify potential predictors of reduction in PaCO_2_ (Table 5). The predictors that were statistically significantly correlated with a reduction in PaCO_2_ were the set flow (*p* < 0.0046), lactates (*p* < 0.031), FEV_1_ (*p* < 0.049), and total hours of use (*p* < 0.015).

## 4. Discussion

HFNC treatment offers several advantages over conventional oxygen delivery systems [34] and was associated with reducing moderate to severe exacerbations in patients with chronic hypoxemic and hypercapnic respiratory failure secondary to COPD. It has also been associated with improvements in quality of life [4,35,36,37]. A systematic review by Bonnevie et al. evaluated HFNC’s effects across various clinical settings, reporting short- and long-term reductions in pCO_2_, enhancements in quality of life—as measured by the St. George’s Respiratory Questionnaire—and a decrease in exacerbation rates at one year. However, no significant changes were observed in exercise capacity or mortality [38]. A key mechanism underlying HFNC’s benefits is the washout of nasopharyngeal dead space, which prevents re-breathing of CO_2_, thereby enhancing alveolar ventilation and reducing pCO_2_ [39]. This effect is facilitated by nasal cannulas that do not occlude the nostrils, allowing expelled gases to be actively cleared. Additionally, HFNC reduces inspiratory muscle effort by delivering gas flows that match or exceed the patient’s peak inspiratory flow (PIF), generating an extrinsic positive end-expiratory pressure (PEEPe) that counteracts intrinsic PEEP (PEEPi) caused by expiratory airflow obstruction in COPD [40,41]. As a result, the threshold load to overcome to trigger the inspiratory airflow is decreased, reducing the work of breathing. Supporting these findings, Nagata et al. demonstrated that long-term HFNC use was associated with a reduction in moderate to severe exacerbation rates and improvements in physiological parameters such as ABG, lung function and quality of life [31]. At the same setting, Storgaard et al. observed significant benefits over 12 months, including quality of life measured and reduced exacerbation-related morbidity [42].

Regarding the short-term effects, previous studies have confirmed that brief HFNC use reduces pCO_2_ levels [43,44,45], attributable to dead space washout and improved mucociliary clearance facilitated by humidified airflow [46]. However, the use of HFNC produces positive airway pressure, which could have significant clinical benefits in terms of improving oxygenation, ventilation, and perfusion matching, decreasing airway resistance, and regulating intrinsic positive end-expiratory pressure (PEEP) [47,48]. HFNC therapy may serve as an effective long-term treatment for stable hypercapnic COPD patients, due to its comfort and its effectiveness to reduction PaCO_2_. Compared to long-term oxygen therapy (LTOT), prolonged HFNC therapy (lasting between 4 and 52 weeks) has been associated with improved health-related quality of life scores and a significant reduction in COPD exacerbations, a result consistently observed across multiple studies [26,31,49].

However, the effects of HFNC are influenced by patient characteristics. Systemic inflammation in COPD concerns the activation of several pathways and plays a crucial role in disease progression and the development of exacerbations [50,51,52]. In our cohort, patients with lower BMI and fewer airway obstructions responded more favorably, exhibiting greater decarbonization and respiratory muscle improvements. A higher BMI can increase diaphragmatic workload, especially when airway obstruction and dynamic hyperinflation (reflected by higher PEEPi) are present, leading to early muscle fatigue and shallow, rapid breathing patterns. These factors diminish the potential benefits of HFNC [53,54].

Our study aimed to evaluate, in a real-world setting, which factors—including anthropometric parameters, respiratory function measures, and HFNC usage—most influenced decarbonization, defined as improved ventilatory efficiency and a reduction in annual exacerbation rates at 3, 6, and 12 months. To assess the impact of each variable on the risk of exacerbations, we conducted a predictive analysis. Secondary objectives included evaluating the longitudinal stability of PaCO_2_ and blood lactate reductions and changes in respiratory function parameters (FEV_1_, FVC, TLC, RV, DLCO, MIP, and MEP). Blood lactate was considered an exploratory metabolic parameter associated with changes in ventilatory status; however, given the potential influence of comorbidities on lactate metabolism, lactate variations should be interpreted cautiously. In our population, patients using HFNC for longer durations at higher flow rates experienced fewer exacerbations—specifically one moderate exacerbation in this group, compared to eight in the group with lower flow and shorter usage. Overall, 12 moderate exacerbations were recorded, with no severe events. This discrepancy highlights the importance of both flow rate and total daily usage hours.

In the higher flow group (>50 L/min), we observed a significant reduction in pCO_2_ from 58.5 mmHg at baseline (T0) to 48 mmHg at 12 months (T12), with a statistically significant decrease already evident at 3 months. Conversely, the lower flow group (<50 L/min) experienced a less pronounced reduction—from 57.23 mmHg at T0 to 52.85 mmHg at T12. Similar patterns were noted for lactate levels, which significantly decreased in the higher flow group (from 1.6 mmol/L to 1.2 mmol/L) at T3; the trend was maintained through T12.

The association between lower BMI and greater PaCO_2_ reduction suggests that body habitus and baseline respiratory mechanics may influence the clinical response to HFNC. Excess body weight has been previously associated with altered breathing patterns and reduced ventilatory efficiency in COPD [55,56]; nevertheless, in the absence of direct measurements of respiratory mechanics, inspiratory effort, or dynamic hyperinflation, no mechanistic conclusions can be drawn from the present data. Similarly, higher tolerated HFNC flow rates and longer daily usage were associated with greater reductions in PaCO_2_ and fewer exacerbations. While previous physiological studies suggest that higher flow rates may enhance ventilatory efficiency through mechanisms such as dead space washout or low-level positive airway pressure [57], these effects were not directly measured in our cohort and should be regarded as plausible hypotheses rather than demonstrated mechanisms. The present study has several limitations: (1) the relatively small sample size, which may affect the stability of the multivariate regression models, with a potential risk of overfitting, and (2) the retrospective observational design and the absence of a control group limit causal interpretation of the findings; therefore, the observed improvements cannot be definitively attributed to HFNC therapy and should be confirmed in prospective controlled studies. Accordingly, these analyses should be interpreted with caution and regarded as exploratory.

## 5. Conclusions

In conclusion, previous studies have shown that long-term use of HFNC has the potential to reduce PaCO_2_ and acute exacerbation, and improve quality of life in patients with stable COPD. HFNC oxygen therapy is a promising technique that is changing the management of patients with chronic respiratory failure. The delivery of heated and humidified oxygen at high-flow rates has several positive effects on the airways and respiratory function. Moreover, the use of a comfortable nasal cannula with this device makes it generally well-accepted by patients. In our study, treatment with HFNC represents a reasonable therapeutic choice to reduce AEs-COPD and reduce PaCO_2_ and lactates in stable COPD patients.

## Figures and Tables

**Table 1:** Baseline characteristics of the study population. Baseline pulmonary function test results are provided, including FEV_1_, FVC, FEV_1_/FVC ratio, total lung capacity (TLC), residual volume (RV), diffusing capacity for carbon monoxide (DLCO), maximal inspiratory pressure (MIP), and maximal expiratory pressure (MEP). Arterial blood gas (ABG) and high-flow nasal cannula (HFNC) settings are described, indicating temperature and flow rates used among the patients.

	Characteristic T0	*n* = 40
**Arterial Blood Gas (ABG)**	**pH**	
	7.35–7.39	27 (68%)
	7.40–7.43	13 (33.5%)
	**pCO_2_**	58.5 (56.5, 61.5)
	**PO_2_**	53.00 (51.50, 55.00)
	**HCO_3_^−^**	34.50 (32.00, 37.00)
	**Lactates**	1.60 (1.40, 1.90)
	**P/F**	252 (245, 261)
**Baseline** Pulmonary **Function Test**	**FEV_1_**	0.73 (0.56, 1.02)
	**FEV_1_%**	42 (28, 51)
	**FVC**	2.06 (1.42, 2.61)
	**FVC**%	70 (60, 78)
	**FEV_1_/FVC**	40 (32, 49)
	**TLC**	6.13 (5.38, 6.85)
	**TLC**%	111 (102, 125)
	**RV**	3.90 (3.35, 4.65)
	**RV**%	182 (169, 200)
	**DLCO**	3.63 (2.71, 4.00)
	**DLCO**%	53 (43, 58)
	**MIP**	4.82 (3.37, 5.21)
	**MIP**%	61.1 (58.5, 64.0)
	**MEP**	6.90 (5.24, 9.64)
	**MEP**%	74.2 (71.7, 76.0)
**High-Flow Nasal Cannula (HFNC) settings**	**HFNC—Temperature**	
	31	15 (38%)
	34	17 (43%)
	37	8 (20%)
	**HFNC—Flow**	
	<40 L/min	12 (31%)
	41–49 L/min	1 (2.5%)
	>50 L/min	22 (68%)
	**HFNC—FiO_2_**	26.50 (25.00, 29.00)
	**Exacerbation in one year**	Moderate–Severe
	0	0 (0%)–20 (50%)
	1	6 (15%)–14 (35%)
	2	19 (48%)–5 (13%)
	3	13 (33%)–1 (2.5%)
	4	2 (5%)–0 (0%)

**Table 2:** Pairwise within-subject comparisons between baseline and follow-up time points (paired Wilcoxon test, BH-adjusted *p*-values).

Variables	T0 vs. T3	T0 vs. T6	T0 vs. T12
pCO_2_	<0.001	<0.001	<0.001
Lactates	<0.001	<0.001	<0.001
pH	0.002	0.017	<0.001
HCO_3_^−^	0.002	<0.001	<0.001
MIP%	0.008	0.004	<0.001
MEP%	0.042	0.017	0.001

**Table 3:** Multivariate analysis evaluating the association between treatment response after 12 months of HFNC therapy and patients’ BMI values. The dependent variables include changes in pCO_2_, lactate levels, FEV_1_, MIP and MEP at 12 months (T12). The analysis shows that BMI is significantly associated with changes in pCO_2_ (*p* < 0.0001), FEV_1_ (*p* = 0.0204), and lactate levels (*p* = 0.0608). Coefficients, standard errors, t-ratios, and *p*-values are reported.

Variables	Coefficient	Standard Error	t-Ratio	*p*-Value
T12_pCO_2_	0.541757	0.116248	4.6603	**<0.0001**
T12_LACTATES	−5.43013	2.80304	−1.9372	0.0608
T12_FEV_1_	0.0895282	0.0368479	2.4297	0.0204
T12_MIP	0.100244	0.131398	0.7629	0.4506
T12_MEP	−0.0621883	0.124223	−0.5006	0.6198

**Table 4:** Multivariate analysis evaluating the association between hours of HFNC use after 12 months of therapy and variation in pCO_2_ and MIP values and number of exacerbations. The dependent variables include changes in pCO_2_, lactate levels, FEV_1_, MIP and MEP at 12 months (T12). Coefficients, standard errors, t-ratios, and *p*-values are reported.

	Coefficient	Standard Error	t-Ratio	*p*-Value
T12_pCO_2_	−0.103389	0.057057	−1.8120	**0.0486**
T12_LACTATES	−1.56128	1.37579	−1.1348	0.2642
T12_FEV_1_	−0.0106254	0.0180857	−0.5875	0.5606
T12_MIP	0.171079	0.0644929	2.6527	**0.0119**
T12_MEP	0.0563721	0.0609711	0.9246	0.3615
EXACERBATION	3	1.66224	1.8048	**0.0488**

**Table 5:** Multivariate analysis evaluating the association between predictors of reduction in PaCO_2_ after 12 months of HFNC therapy. Coefficients, standard errors, t-ratios, and *p*-values are reported.

	Coefficient	Standard Error	t-Ratio	*p*-Value
T12_LACTATES	4.51951	1.99058	2.2704	**0.0301**
T12_FEV_1_	−0.0492924	0.0265987	−1.8532	**0.0491**
T12_MIP	0.11602	0.104833	1.1067	0.2767
T12_MEP	−0.132716	0.105866	−1.2536	0.2191
T12_HOURSE OF USE	−0.843203	0.330452	−2.5517	**0.0157**
T12_RV	0.0116039	0.0154765	0.7498	0.4589
FLOW	0.0566292	0.108287	2.5230	**0.0046**

## Data Availability

Data are contained within the article.
